# Transcriptome Analysis of *Lycoris chinensis* Bulbs Reveals Flowering in the Age-Mediated Pathway

**DOI:** 10.3390/biom12070899

**Published:** 2022-06-27

**Authors:** Fengjiao Zhang, Guanghao Cheng, Xiaochun Shu, Ning Wang, Zhong Wang

**Affiliations:** 1 Institute of Botany, Jiangsu Province and Chinese Academy of Sciences, Nanjing 210014, China; zfjiao@yeah.net (F.Z.); chengghao@yeah.net (G.C.); sxc@cnbg.net (X.S.); wangning813@njau.edu.cn (N.W.); 2 Jiangsu Key Laboratory for the Research and Utilization of Plant Resources, Jiangsu Provincial Platform for Conservation and Utilization of Agricultural Germplasm, Nanjing 210014, China

**Keywords:** *Lycoris chinensis*, transcriptome, flowering, bulb age, hormone signaling

## Abstract

*Lycoris* is a summer bulbous flower that commonly needs to go through a long period of vegetative growth for 3 to 5 years before flowering. Plant flowering is regulated by a complex genetic network. Compared with most perennial flowers, knowledge on the molecular mechanism responsible for floral transition in bulbous flowers is lacking, and only a few genes that regulate flowering have been identified with few reports on the floral transition in *Lycoris*. In this study, we identified many differentially expressed genes (DEGs) and transcription factors (TFs) by RNA-Seq in *L. chinensis* bulbs of different ages, including one- to four-year-old nonflowering bulbs and four-year-old flowering bulbs. Some DEGs were enriched in Gene Ontology (GO) terms between the three- and four-year-old bulbs, and there most genes were enriched in terms of metabolic process and catalytic activity. In the four-year old bulbs, most of the DEGs that may be involved in flowering were classified under the GO term biological process, which was a totally different result from the vegetative bulbs. Some DEGs between flowering and nonflowering bulbs were enriched in plant hormone signal transduction, including the hormones auxin, cytokinin, abscisic acid, and ethylene, but no DEGs were enriched in the gibberellin pathway. Auxin is the main endogenous phytohormone involved in bulb growth and development, but cytokinin, abscisic acid, and ethylene were shown to increase in flowering bulbs. In addition, energy-metabolism-related genes maintain a high expression level in large bulbs, and some positive regulators (SPL, COL, and AP1) and early flowering genes were also shown to be highly expressed in the meristems of flowering bulbs. It suggested that sugar molecules may be the energy source that regulates the signal transduction of flowering by connecting with phytohormone signaling in *Lycoris*. A total of 1911 TFs were identified and classified into 89 categories, where the top six families with the largest gene numbers were C2H2, NAC, AP2/ERF-ERF, C3H, MYB-related, and WRKY. Most DEGs were in the AP2/ERF-ERF family, and most of them were downregulated in 4-year-old flowering bulbs. A number of families were reported to be involved in plant flowering, including NAC, AP2/ERF, MYB, WRKY, bZIP, MADS, and NF-Y. These results can act as a genetic resource to aid in the explanation of the genetic mechanism responsible for the flowering of *Lycoris* and other bulbous flowers.

## 1. Introduction

The lifecycle of angiosperms includes four stages: embryo/juvenile, adulthood, flowering and reproduction, and senescence [1]. Flowering, an important stage in the lifecycle of plants, is not only beneficial for plant reproduction and species evolution, but is also a prerequisite for crop seed or fruit harvesting and is even an important trait of ornamental plants. Plant flowering is a process from flower formation induction to flower initiation, and flower organogenesis. Flower formation induction is the stage at which a plant transforms from vegetative to reproductive growth, and it determines the flowering time of the plant [2]. It is regulated by a complex gene network that integrates a variety of exogenous environmental factors (such as light and temperature) and plant endogenous factors (such as plant hormones and age) to ensure an accurate flowering time [3].

Previous genetic and molecular studies on the model plant *Arabidopsis thaliana* showed that there are five main pathways that regulate plant flowering: the photoperiod, vernalization, age, GA, and the autonomous pathway [4]. For example, the photoperiod pathway regulates *FLOWERING LOCUS T* (*FT*) and *SUPPRESSOR OF OVEREXPRESSION OF CO1* (*SOC1*) through *CONSTANS* (*CO*) to upregulate expression and promote flowering. The interaction of *CO* and different proteins is very important for regulating the accurate transcription of downstream genes. Vernalization and autonomous pathways inhibit the activity of *FLOWERING LOCUS C* (*FLC*) and increase the expression of *FT* and *SOC1*, thereby promoting flowering [5]. In the aging pathway, miR156 and its target genes, *SQUAMOSA PROMOTER BINDING PROTEIN-LIKE* (*SPL*) and miR172, are key components of this pathway, regulating the transition from plant vegetative growth to reproductive development [6]. From the juvenile to the adult stage, the expression level of miR156 gradually decreases, and the expression of the negative regulatory target *SPL* increases, which promotes an increase in miR172 expression; miR172 downregulates the flower-forming inhibitor *APETALA2* (*AP2*) and promotes the transcription of flower-forming integrins (*FT*, *SOC1,* and *AGL24*) and floral meristem recognition genes (*FUL*, *LFY*, *AP1*), finally inducing plant flowering [7]. Under the single or combined action of these pathways, the upstream genes precisely regulate the expression of the two key downstream flowering genes, *FT* and *SOC1*, at the transcription and translation levels, thereby affecting flowering.

In these regulatory pathways, some genes show relative conservation in the regulation of flowering in perennials, such as SOC1 and FT, which are among the key transcription factors that integrate signals from almost all genetic pathways. The key genetic regulators of floral transition have been well characterized in model species, and increasing numbers of related genes are being identified in crops [8,9]. However, the main regulatory pathways are varied in different species. For example, many annual herbaceous species germinate in the autumn and over winter and then flower in spring, having typical light and temperature responses during the flowering initiation stage [10]. In these taxa, low-temperature vernalization and long-day photoperiods are often required, which helps to relieve repressors of flowering and activate floral inducers [11]. However, in many perennials, such as most woody fruit trees [12,13] and bulb flowers [14,15], the acquisition of competence to flower is usually strongly delayed due to the high dependency of the miR156/SPL system [16], where the age pathway is the main factor that regulates flowering.

Bulbous flowers are rich in variety with strong stress resistance and are often used as garden flowers, cut flowers, and pot flowers. Similar to other perennial flowers, bulbous flowers also need to undergo a transition from vegetative growth to reproductive development before they can bloom. However, due to the bulb size and environmental factors, most bulbous flowers often take several years to flower [17]. Having a long vegetative growth period leads to higher production costs. *Lycoris* is a genus containing about 20 species of bulb-producing perennial plants from the family Amaryllidaceae, which are native to eastern and southern Asia and are mainly distributed in China and Japan [18]. They bloom from summer to autumn, and then the leaves grow in autumn, winter, and spring. The flowers have a special spidery shape and rich colors. In addition, their bulbs are abundant in Amaryllidaceae alkaloids. Among these, the component galantamine is used in medications to treat Alzheimer’s-type dementia [19,20]. Thus, they have both high ornamental and medicinal value. However, it takes a long time for them to transition from seed to bloom, often as long as 3–5 years, so they are often propagated by the division of bulbs. It also takes 2–3 years of vegetative growth for the small asexually reproduced bulbs to complete the flower transformation process [21], which not only slows the breeding process of *Lycoris*, but also increases the production cost of commodity bulbs. Therefore, it is important to explore the factors that regulate the flowering transition of *Lycoris*.

Several environmental factors that may affect the flowering of *Lycoris* have been focused on in previous research, such as the low temperature [22], moderate shade, and water requirement [23]. These studies involved physiological and biochemical measurements, and provide some evidence that these endogenous and exogenous factors can promote or inhibit the flowering of *Lycoris* to a certain extent [22,23,24]. For example, earlier heating may lead to earlier flower bud development in spring [22], long days and drier environments increase reproductive growth, and moderate shade improves the yield and quality of cultivated *L. radiata* flowers [23]. However, it needs to be mentioned that all of these studies were based on bulbs with the ability to flower. The large *Lycoris* bulbs with flowering ability commonly experience a period of summer dormancy and then flower without leaves. The exogenous environmental factors may have some effect on the flowering time, but they are not the key genetic factors that determine whether the bulbs can flower or not. The main genetic factors that affect bulb development, apical meristems on flowering, and floral transformation are largely unknown. In the present study, we aimed to reveal the differentiation of gene expression and explore the key regulatory genes involved in the process of bulb development and flowering by high-throughput RNA sequencing (RNA-Seq) technology and to study the molecular regulation mechanism involved in flowering at the molecular level in *Lycoris*. The results provide abundant genetic resources that can be used in research on the flowering regulation of *Lycoris* and other bulbous flowers.

## 2. Materials and Methods

### 2.1. Plant Material and Sample Collection

One- to four-year-old *L. chinensis* bulbs were used in this study. Some four-year bulbs have the ability to flower, and some do not, and these were collected separately in this study. The bulbs were planted in the Nanjing Botanical Garden Mem. Sun Yat-Sen (Nanjing, China). The 1–4-year-old bulbs were selected (P1–P5) and cut along the middle of the bulb using a scalpel, and then the shoot apical meristem (SAM) was carefully cut off. Samples P1–P5 were 1–3-year-old bulbs, 4-year-old nonflowering bulbs and 4-year-old flowering bulbs. Fifteen meristem samples (3 replications for each sample) were collected and immediately frozen in liquid nitrogen and stored at −80 °C for RNA-Seq and qRT-PCR validation analyses.

### 2.2. RNA Extraction, Library Construction and Transcriptome Sequencing

Total RNA from 15 meristem samples was extracted using the Quick RNA Isolation Kit (Huayueyang Biotechnology Co., Ltd., Beijing, China) following the manufacturer’s protocol. The purity and quality of RNA were detected by 1.0% (*w*/*v*) agarose–gel electrophoresis, and the quality and quantity were further measured using the Agilent 2100 bioanalyzer. Then, the cDNA libraries for Illumina sequencing were constructed by mRNA enrichment, double-stranded DNA synthesis, sequencing adapter modification and fragment size selection, PCR amplification, and library detection. The qualified libraries were sequenced on the Illumina HiSeq2500 platform at Novogene Bioinformatics Technology Co., Ltd. (https://www.novogene.com/, accessed on 20 January 2018, Tianjin, China). Raw reads were deposited in the NCBI database (https://www.ncbi.nlm.nih.gov/, accessed on 8 June 2022) under BioProject number PRJNA847051 (SRA accession: SRR19578267–SRR19578281).

### 2.3. Raw Data Processing, Transcriptome Assembly, and Gene Functional Annotation

In order to fit the information analysis, the raw reads were processed by removing the adaptor sequences. Reads with a ratio of more than 0.1% N (N means that the base information cannot be determined) and the low-quality reads were removed. Then, the clean reads were assembled de novo by Trinity software (r20140413p1, http://trinityrnaseq.github.io, accessed on 30 January 2018, Broad Institute and Hebrew University of Jerusalem) [25]. The longest transcript sequence of each gene was taken as the unigene for subsequent analysis. For the functional annotation of all the unigenes, BLASTx (E-value ≤ 10^–5^) was carried out on seven major public databases, including the Nr (NCBI nonredundant protein sequences), Nt (NCBI nucleotide sequences), Pfam (Protein family, http://pfam.sanger.ac.uk/, accessed on 30 January 2018), KOG (euKaryotic Ortholog Groups), Swiss-Prot (A manually annotated and reviewed protein sequence database, http://www.ebi.ac.uk/uniprot/, accessed on 30 January 2018), KEGG (Kyoto Encyclopedia of Genes and Genomes, http://www.genome.jp/kegg/, accessed on 30 January 2018), and GO (Gene Ontology, http://www.geneontology.org/, accessed on 30 January 2018). The GO and KEGG annotated genes were classified. Then, the GO annotated genes were classified into three major categories (BP, biological process; CC, cellular component; MF, molecular function). KOG is divided into 26 groups, and the KO annotated genes were classified according to the KEGG metabolic pathways they participate in. The iTAK program and database were used to identify the transcription factors [26].

### 2.4. Differentially Expressed Gene (DEG) Analysis

The clean reads from each sample were mapped to the reference sequence assembled by Trinity using RSEM software [27] with the bowtie2 default parameter mismatch 0. According to the statistics of the bowtie comparison, the readcount number of each sample mapped to each gene was obtained, and further FPKM values were conversed and the gene expression level was analyzed. The readcount was input for the differentially expressed gene (DEG) analysis. In this study, three replications of each sample were used. We used DESeq [28] for the DEGs analysis with a screening threshold of padj < 0.05. When the log_2_FoldChange of samples was more than 1.5, it was screened for DEG analysis. In order to better understand the function of DEGs, we performed GO and KEGG enrichment analyses for each comparison. GOseq [29] was used for the GO enrichment analysis, and the number of significantly enriched genes in each GO term was displayed in the form of a histogram. The KEGG enrichment analysis was performed by KOBAS (2.0) with the parameters -fdr to BH and FDR ≤ 0.05. The 20 most enriched pathway entries are displayed in a scatter plot. If there are less than 20 enriched pathway entries, all pathway entries were displayed. In addition, the pathway maps are also shown for the enriched differential genes. The clustered heat maps were drawn using Tbtools software [30] with a log2 scale.

### 2.5. qRT-PCR Validation

To validate the accuracy and reliability of the transcriptome results, 20 DEGs involved in flowering were randomly selected for the quantitative real-time polymerase chain reaction (qRT-PCR), and *EXP1* was selected as the internal standard control [31]. The gene-specific primers for qRT-PCR (Table S1) were designed using Primer3 Release 2.3.4 [32] (pp. 365–386) and synthesized by General Biosystems Co., Ltd. (Chuzhou, China). qRT-PCR was conducted on a StepOnePlus™ real-time PCR system (Applied Biosystems, Foster City, CA, USA). The first cDNA strand was synthesized with the PrimeScript^TM^ reagent kit (Takara, Dalian, China). The mRNA expressions were quantified using the SYBR Premix Ex Taq II kit (TaKaRa, Dalian, China). Three biological and technical replicates for each gene were employed. The mean ± standard deviation of three biological replicates for each sample was calculated. The relative RNA expression levels were analyzed using the 2^−ΔΔCT^ method [33]. The STDEVA function in Excel was used to assess the standard errors of deviation.

## 3. Results

### 3.1. Overview of Transcriptome Sequencing Data Analysis

**Figure 1:**
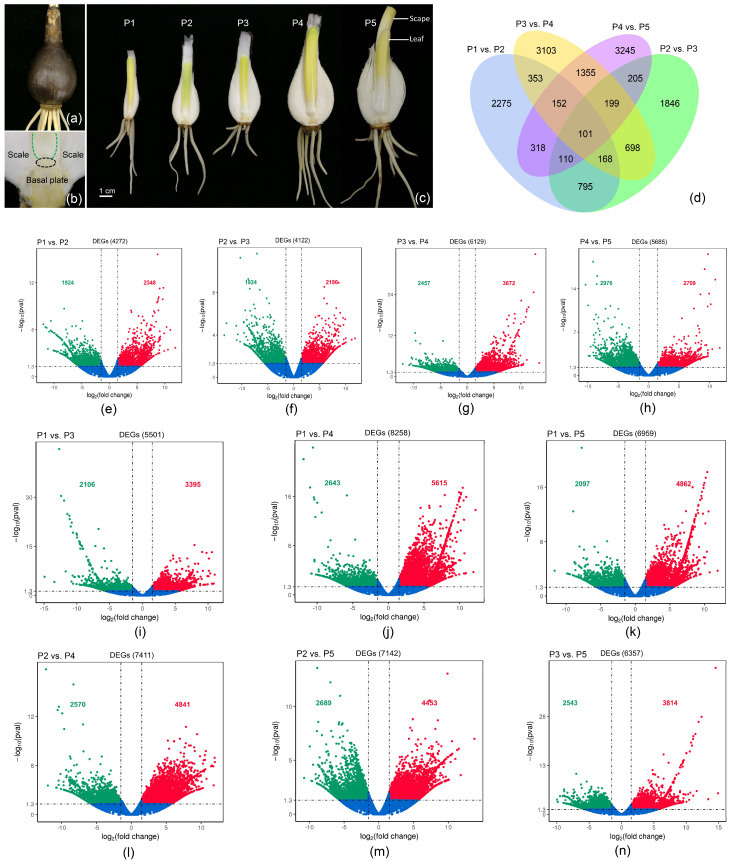
The samples subjected to RNA-Seq and the numbers of differentially expressed genes (DEGs) for the samples: (**a**) external morphology of the bulb; (**b**) sectional view of the internal organizational structure of the bulb (the samples were collected from the meristem site); (**c**) morphological characteristics of the five samples. P1−P3: 1−3-year-old bulbs that cannot flower, P4−P5: 4-year-old nonflowering and flowering bulbs, respectively. (**d**) Venn diagram of the DEGs in combinations of P1 vs. P2, P2 vs. P3, P3 vs. P4, and P4 vs. P5. (**e**–**n**) The volcano plots show the number of DEGs for each combination of different samples.

In order to detect the key factors that regulate flowering in bulbs of different ages in *L. chinensis*, five samples (three biological replicates for each sample) were selected for the construction of 15 cDNA libraries and high-throughput RNA-Seq. They were 1–3-year-old bulbs that cannot flower, and 4-year-old bulbs, both nonflowering and flowering (Figure 1a–c, P1–P5). A summary of the RNA-Seq results is shown in Table 1. In each library, around 50 million clean reads were generated and about 60% of the reads were mapped in reference sequences. For each library, 7.0 Gb of data were obtained. The Q30 percentages for all libraries exceeded 91.5%, and the GC (Guanine and Cytosine) contents ranged from 45.65% to 47.05%. A total of 200,902 unigenes were identified (Table S2). To identify the differentially expressed genes affecting bulb development and flowering regulation, we compared the global transcriptomic profiles of five samples (P1–P5). The Venn diagram in Figure 1d shows the number of DEGs obtained from four group comparisons, where 101 genes were commonly and differentially expressed during the five stages. During the total vegetative growth stage (P1–P3), the number of DEGs was about 4000, of which upregulated and downregulated genes accounted for about half of these (Figure 1e,f, Table S3). During the transition from vegetative growth to reproductive growth (P3 to P4 and P4 to P5), the number of DEGs increased significantly, reaching more than 5500 (Figure 1g,h, Table S3). The volcano plots for all combinations are shown in Figure 1e–n.

### 3.2. Gene Function Annotation

**Figure 2:**
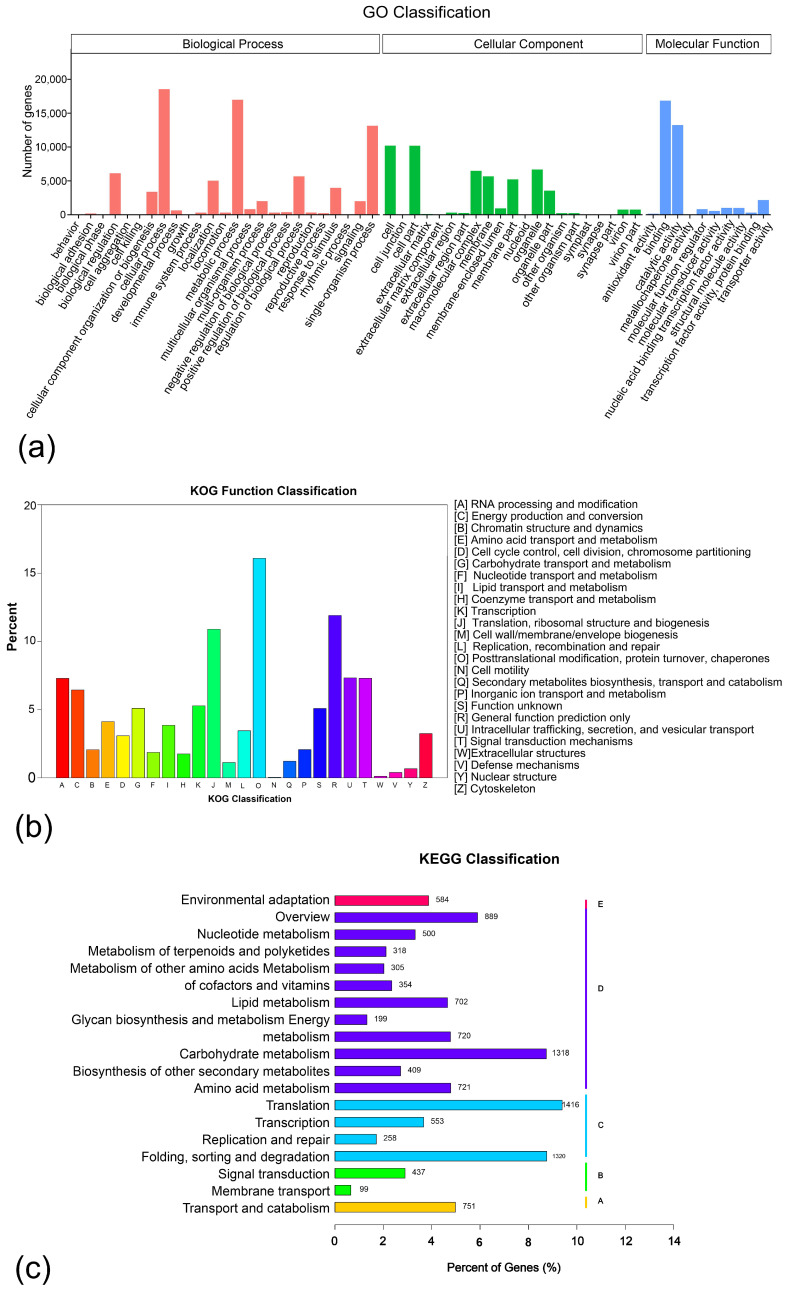
The annotation of identified unigenes in three databases. (**a**) GO function classification, where the horizontal axis shows the three GO terms: biological processes, cellular components, and molecular functions; the vertical axis shows the number of genes annotated under each term. (**b**) KOG function classification, where the vertical axis gives the names of the 26 KOG groups, and the horizontal axis gives the percentages of annotated genes. (**c**) KEGG pathway annotation, where the vertical axis gives the names of the KEGG metabolic pathways, and the horizontal axis gives the numbers and percentages of genes annotated to this pathway.

In order to understand the functions of the unigenes, gene function annotations were conducted in seven major databases, including Nr, Nt, Pfam, KOG/COG, Swiss-Prot, KEGG, and GO. A total of 29.62% of them were annotated in at least one database, and the percentage of annotated gene functions in NR, NT, GO, PFAM, and Swiss-Prot was 22.71, 10.34, 17.21, 17.05, and 15.04, respectively (Table 2). After annotation of GO, KOG, and KEGG, the unigenes were categorized into different groups. For GO annotation, three categories were classified: BP (biological process), CC (cellular component), and MF (molecular function). Most unigenes were annotated with the terms ‘cellular process’, ‘metabolic process’, and ‘binding’ (Figure 2a). A total of 25 classifications were annotated with KOG functions. The genes related to ‘posttranslational modification’, ‘protein turnover’, and ‘chaperones’ were the most abundant, accounting for more than 15% of the total number. This was followed by the classifications of ‘general function prediction only’ and ‘translation, ribosomal structure, and biogenesis’, which accounted for more than 10% of the total genes. The least common categories were ‘cell motility’, ‘extracellular structures’, and ‘defense mechanisms’ (Figure 2b). After the KOG annotation, the genes were classified by KEGG according to the metabolic pathways they participate in. They genes were found to be involved in a total of 19 pathways. Of them, the ‘translation’ pathway enriched the largest number of genes, followed by the ‘folding, sorting, and degradation’ and ‘carbohydrate metabolites’ pathways. The number of genes involved in these three classifications was more than 1300, which is significantly higher than that in other pathways (Figure 2c).

### 3.3. GO and KEGG Enrichment of Differentially Expressed Genes (DEGs)

**Figure 3:**
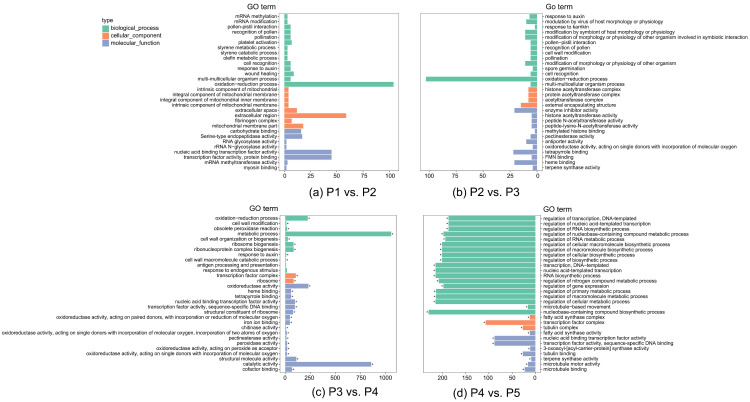
GO enrichment of DEGs in different comparisons. (**a**–**d**) the comparison of P1 vs. P2, P2 vs. P3, P3 vs. P4, and P4 vs. P5. P1−P3: 1−3-year-old bulbs that cannot flower, P4−P5: 4-year-old nonflowering and flowering bulbs. The vertical axis is the names of the GO terms; the horizontal axis is the numbers of genes enriched in each term. The three GO term types are shown in different colors. Asterisks represent significant enrichment.

The GO enrichment of DEGs identified the biological functions significantly related to these genes. In the pairwise comparison enrichment analysis, enriched GO terms were shown in bulbs aged more than three years, including comparisons for P3 vs. P4 and P4 vs. P5 (Figure 3c,d). These pairs had significantly more genes than P1 vs. P2 and P2 vs. P3, where the number of DEGs in most GO terms was less than 25, and most genes were enriched in the oxidation–reduction process (Figure 3a,b). During the transition from vegetative growth to reproductive growth, most genes were classified by the GO term molecular function and two processes, metabolic processes (biological process), and catalytic activity (molecular function), which were enriched in more than 700 genes (Figure 3c). In four-year-old bulbs, most genes that determine whether to flower were classified under the GO term biological process, and these terms were totally different from the DEGs identified in the other three comparisons (Figure 3d).

**Figure 4:**
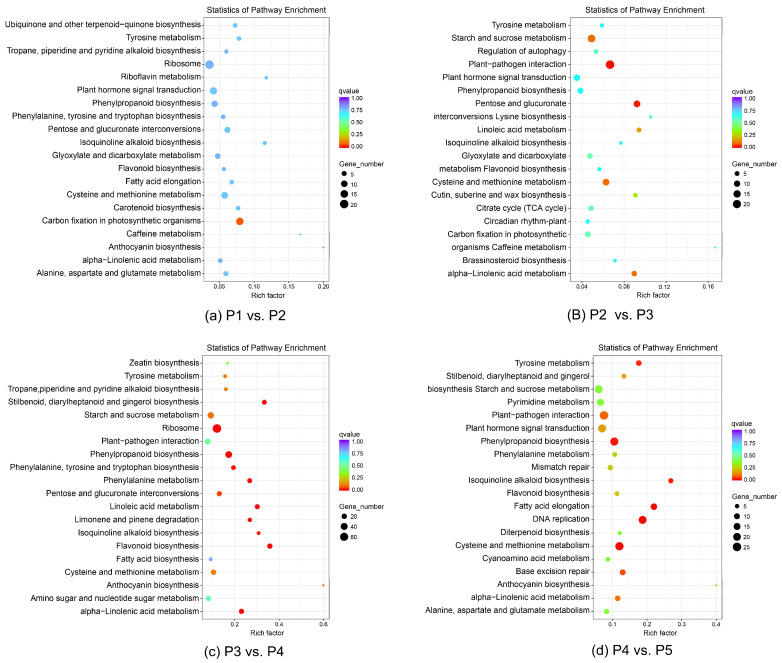
Top 20 enriched KEGG pathways for the DEGs in different comparisons. (**a**−**d**) the comparison of P1 vs. P2, P2 vs. P3, P3 vs. P4, and P4 vs. P5. P1−P3: 1−3-year-old bulbs that cannot flower, P4−P5: 4-year-old nonflowering and flowering bulbs. The vertical axis represents the names of the pathways, and the horizontal axis represents the Rich factor corresponding to the pathways. The q-value is represented by the color of the dot; the smaller the q-value, the closer the color is to red. The number of DEGs of each pathway is represented by the size of the dot.

Through significant pathway enrichment, the most important biochemical metabolic pathways and signal transduction pathways involving the differentially expressed genes were determined. For each comparison, a total of 20 pathways were enriched and the assigned DEG numbers were 346 (P1 vs. P2), 301 (P2 vs. P3), 750 (P3 vs. P4), and 543 (P4 vs. P5). There were six pathways that were simultaneously enriched in four comparisons: ‘Tyrosine metabolism’, ‘Phenylpropanoid biosynthesis’, ‘Isoquinoline alkaloid biosynthesis’, ‘Flavonoid biosynthesis’, ‘Cysteine and methionine metabolism’, and ‘alpha-Linolenic acid metabolism’. This suggests the universality of these pathways in bulb development, biosynthesis, and metabolism (Figure 4). In two- to four-year-old bulbs, the DEGs were enriched in two common pathways: ‘starch and sucrose metabolism’ and ‘plant–pathogen interaction’ (Figure 4b–d). Two pathways ‘Stilbenoid, diarylheptanoid, and gingerol biosynthesis’ and ‘Phenylalanine metabolism’ were enriched in the stage from vegetative growth to reproductive growth (P3 vs. P4 and P4 vs. P5) (Figure 4c,d). Several pathways were only enriched in four-year-old nonflowering and flowering bulbs: ‘pyrimidine metabolism’, ‘DNA replication’, ‘diterpenoid biosynthesis’, and base excision repair (Figure 4d).

### 3.4. Key Genes Involved in Bulb Growth and Flowering in L. chinensis

Because of the genetic conservation of plant flowering, many genes have been shown to be involved in different flower-forming pathways and have conserved regulatory functions in model plants. In this study, we classified the key up- and downregulated genes that may be involved in flowering between P4 and P5 samples. In flowering bulbs (P5), there were more upregulated genes than in four-year-old nonflowering bulbs (P4), for example, squamosa promoter-binding-like protein 5 (SPL5), agamous-like MADS-box protein AGL14/AGL61, zinc finger protein CONSTANS-LIKE 15-like, zinc finger CCCH domain-containing proteins, AP2/ERF, energy-metabolism-related genes, histones, hormone-related genes (cytokinin response regulator protein, abscisic acid receptor, indole-3-acetic acid-amido synthetase GH3.1, gibberellin-regulated protein), and some transcription factors. The downregulated genes in P5 were the terminal flower 1-like protein, UPSTREAM OF FLC-like, AP2-11, auxin-responsive and induced protein, ethylene insensitive 3, E3 ubiquitin-protein, and F-box protein and transcription factors (IBH1-like and NAC) (Table 3). Based on 469 DEGs potentially involved in flowering, principal component analysis (PCA) was performed to explore the relationships between gene expression and samples (Figure S1). Results from the PCA analysis were able to distinguish samples of different ages together with flowering and nonflowering bulbs, indicating that the biological replicates of samples were good and the genes involved in bulb development and flowering had specific expression patterns in each sample.

**Figure 5:**
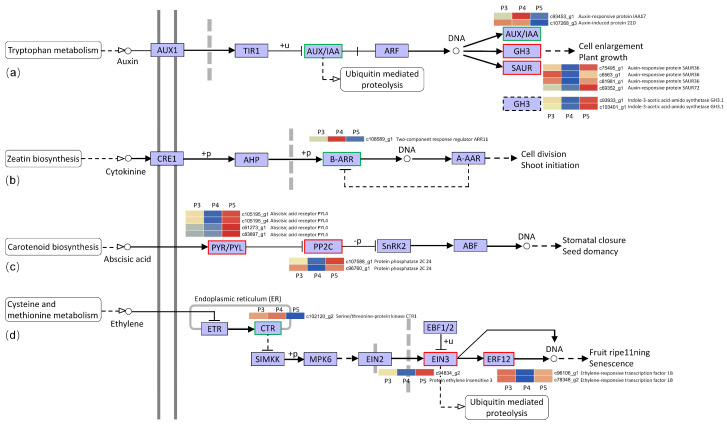
Enriched KEGG pathway terms associated with plant hormone signal transduction in P4 vs. P5. (**a**–**d**) The auxin, cytokinin, abscisic acid, and ethylene signal transduction pathways. The borders of KO nodes of upregulated genes are marked in red, and the downregulated genes are marked in green. The expression level of the related unigenes from P3 to P5 are shown in the heatmap beside the nodes, the color from blue to red means that the gene expression level is from low to high.

Based on the genes identified as being involved in flowering regulation, hormone signaling is considered the main mediating factor in the processes of vegetative to reproductive transformation in plants. In the KEGG enrichment of DEGs, some genes were shown to be enriched in the main pathways of plant hormone signal transduction, including auxin, cytokinine, abscisic acid, and ethylene, but no DEGs were enriched in the gibberellin pathway (Figure 5). In the auxin signal transduction pathway, two unigenes (AUU/IAA), annotated as the auxin-responsive protein IAA and the auxin-induced protein, were downregulated in the flowering bulb. Four unigenes encoding the auxin-response protein SAUR and two unigenes encoding indole-3-acetic acid-amido synthetase GH3 were upregulated in the flowering bulbs. They work together to regulate cell enlargement and plant growth and further affect *Lycoris* flowering (Figure 5a). In the cytokinin signal transduction pathway, one unigene encoding a type-b response regulator (B-ARR) was downregulated in the flowering bulbs, showing the same pattern as unigenes related to AUU/IAA biosynthesis (Figure 5b). In the abscisic acid signal transduction pathway, four abscisic acid receptors PYL4 were upregulated and shown to inhibit protein phosphatase 2C synthesis, but two unigenes of protein phosphatase 2C were also upregulated, showing the negative regulatory function of stomatal closure and seed dormancy. This indicates that abscisic acid may play a negative role in the *Lycoris* floral transition (Figure 5c). In the ethylene biosynthesis pathway, one ethylene-insensitive protein and two ethylene-responsive transcription factors showed higher expression levels in the flowering bulbs, suggesting that they play positive roles in *Lycoris* flowering (Figure 5d).

**Figure 6:**
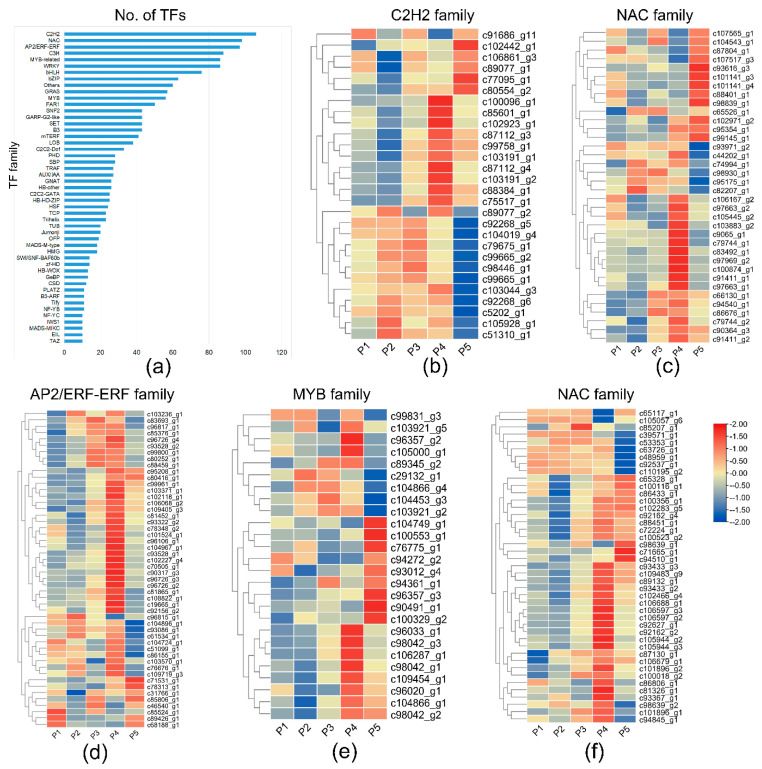
The statistics and abundance of transcription factor families (TFs): (**a**) forty−eight TF families with more than 10 identified unigenes; (**b**–**f**) comparison of P4 and P5 showing the differentially expressed TFs in the C2H2, NAC, AP2/ERF-ERF, MYB-related, and WRKY families.

In addition, transcription factor (TF) genes play various roles in the regulation of plant growth and development and the defense response to adversity. Multiple TFs families were differentially expressed in different samples. Here, we identified 1911 TFs using the iTAK program through the RNA-Seq of *L. chinensis* bulbs. They were classified into 89 categories, and 48 families with more than 10 unigenes are shown in Figure 6a. A number of families were reported to be involved in plant flowering, including NAC, AP2/ERF, MYB, WRKY, bZIP, MADS, and NF-Y. In these families, the top six with the largest numbers of members (more than 80 in each family) were C2H2, NAC, AP2/ERF-ERF, C3H and MYB-related, and WRKY. In the AP2/ERF-ERF family, there were the greatest number of differentially expressed genes in the samples (Figure 6d). The differentially expressed genes in four families other than C3H (C2H2, NAC, MYB-related, and WRKY) are shown in heatmaps (Figure 6b,c,e,f).

### 3.5. qRT-PCR Validation of the RNA-Seq Results

**Figure 7:**
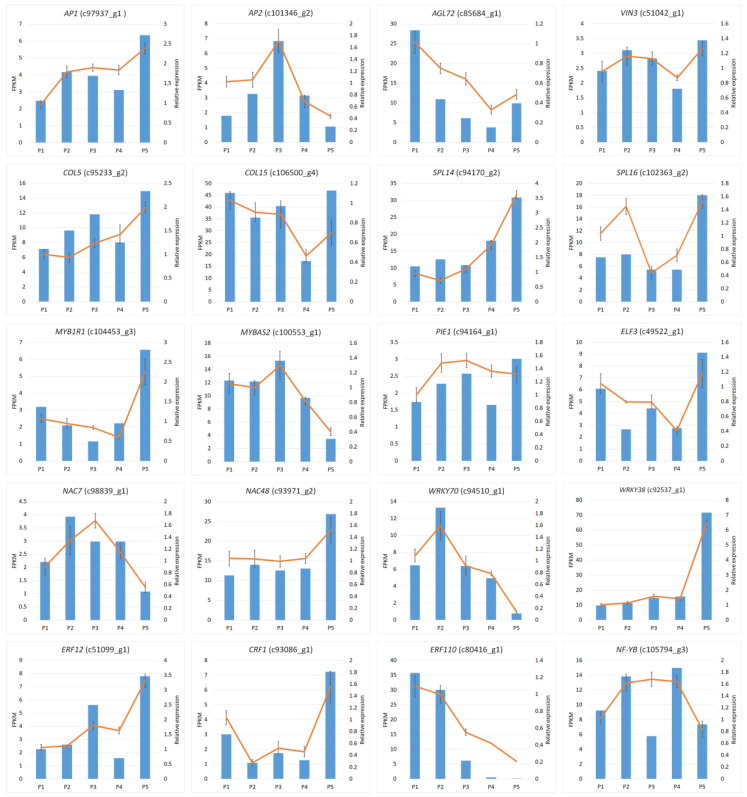
qRT-PCR validation of the expression level of 20 DEGs related to plant flowering. The x-axis shows the five samples, and the y-axis shows the FPKM by RNA-Seq and the relative quantitative expression level by qRT-PCR for each unigene.

In order to explore the main genetic factors related to bulb age and flowering regulation, we summarized the key genes involved in regulating bulb development and the transition from vegetative growth to reproductive development and verified the accuracy of the transcriptome by qRT-PCR. A total of 20 key genes mediating the flowering time were selected for qRT-PCR validation, including *FT*, *CONSTANS-LIKE 1*, *CONSTANS-LIKE 15*, *APETALA1*, *APETALA2, AGL72*, *NF-YB3*, and some genes of the *SPL* family. The expression patterns of the candidate unigenes indicated a high degree of consistency between qRT-PCR and RNA-Seq, indicating the reliability of the RNA-Seq data (Figure 7).

## 4. Discussion

It is well known that the flowering transition of plants is a complex morphological and physiological change that occurs in response to endogenous factors (hormones, age, autonomous, and sugar signals) and exogenous environmental factors (light and temperature). Plants integrate these responses to act on downstream floral integrators to regulate flowering [34,35]. For nonmodel plants and crops without a reference genome, a large amount of RNA-Seq data facilitates gene mining to regulate the plant stress responses, growth, and development [36] (pp. 175–185) [37,38], which also has been widely used to explore the genetic regulation of flowering in many plants, such as bamboo orchid flowering [39] and the flowering transition of saffron [40] and roses [41]. Compared with many perennial flowers, data on gene identification and molecular mechanisms that regulate flowering in bulbous flowers are insufficient, and only a few genes have been identified. For example, *FT* and *LFY* regulate the flowering of *Narcissus tazetta* [42], and *COL* and *FT* regulate the flowering of *Lilium* [43,44]. The reproductive initiation gene *TGSQA* (a homolog of Arabidopsis *APETALA1*) acts as a flowering activator to regulate flowering in *Tulipa gesneriana* [45]. In bulbous flowers, in the process of transitioning from vegetative growth to reproductive development and acquiring the flowering ability, the genes and substances in the meristem at the base of the bulb change as the bulb size increases and flower when the environment is suitable [15]. Therefore, bulb size and age are considered key factors affecting flowering in bulbous flowers. In this study, a total of 200,902 unigenes were assembled from 1–4-year-old vegetative and reproductive *L. chinensis* bulbs, and many transcription factors and key genes that may regulate plant flowering were identified. The result provides genetic information for the flowering regulation in *L. chinensis*.

**Figure 8:**
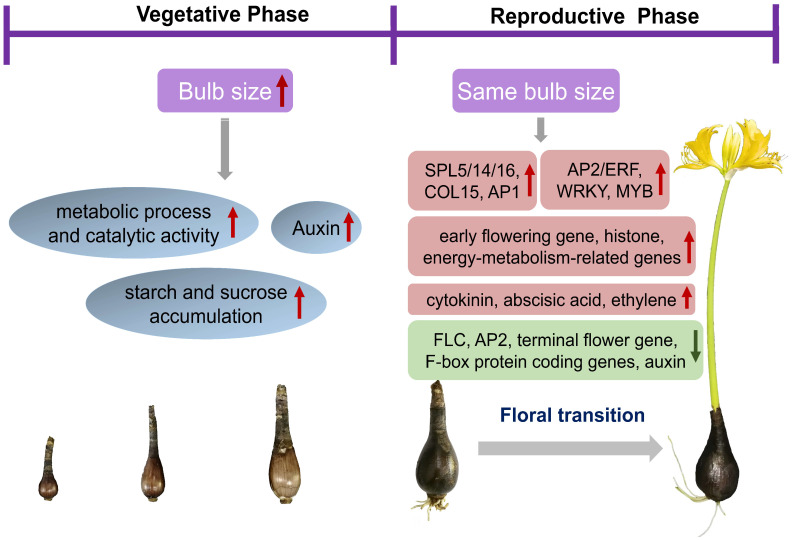
Hypothetical model for the regulatory networks of bulb growth and flowering in *Lycoris chinensis*. Up and red arrows indicate increased or upregulated expression, down and green arrows indicate downregulated gene expression.

In bulbs of different ages, a large number of DEGs were identified. These included many key genes and TFs that may be involved in bulb development and flowering, such as the key up- and downregulated genes between four-year-old nonflowering and flowering bulbs (Table 3). According to the pattern of these DEGs, the overall conclusion is that metabolic processes and energy accumulation are enhanced with increasing age and enlarged bulb size, and auxin is the main endogenous phytohormone involved in bulb growth and development during this process. Once the age and size of bulbs reach the bloom standard, many key genes and factors perform functions to regulate flowering, including positive regulators (*SPL*, *COL*, and *AP1*, high expression in flowering bulbs), negative regulators (*FLC* and *AP2*, terminal flower gene, low expression in flowering bulbs), early flowering genes, and histones. Meanwhile, energy metabolism and hormones also contribute to the floral transition and flowering. The energy-metabolism-related genes maintain high expression levels, and the endogenous phytohormones, such as cytokinin, abscisic acid, and ethylene also increase in flowering bulbs. In contrast, the genes related to auxin synthesis are downregulated (Figure 8). It is suggested that sugar molecules may be the energy sources that regulate flowering signal transduction by connecting with phytohormone signaling in *Lycoris*.

The enrichment analysis of DEGs found that the DEGs were enriched in different KEGG pathways only in the three-year-old and four-year-old bulbs, suggesting that these pathways may be the key pathways involved in the floral transition in *L. chinensis*. From three-year-old to four-year-old nonflowering bulbs, two pathways, ‘metabolic process’ and ‘catalytic activity’, enriched the most DEGs. In the P3 vs. P4 and P4 vs. P5 comparisons, two pathways ’starch and sucrose metabolism’ and ‘anthocyanin biosynthesis’ enriched DEGs, but the DEGs were enriched in the plant hormone signal transduction pathway in the P4 vs. P5 group. Sugar molecules function as energy sources and osmotic regulators and have been recognized as important regulators of floral signal transduction that connect with phytohormone signaling in many plants [46,47,48]. Proteomic studies of the flowering and dormancy of *L. radiata* revealed a large number of differentially expressed proteins involved in the energy and sugar metabolic processes by GO and pathway enrichment [49]. Therefore, our results indicate that there may be active starch and sucrose metabolism, anthocyanin biosynthesis, and catalytic activity in the meristem with the enlargement of the bulbs, and the plant hormone signal is the main factor that induces the floral transition in four-year-old *L. chinensis* bulbs. In the metabolism or response to phytohormones, the gibberellic acid (GA) pathway did not enrich DEGs between two samples of four-year-old bulbs. However, in the auxin, cytokinin, abscisic acid, and ethylene pathways, there were some differentially expressed genes that may affect the flowering regulation of *L. chinensis*; for example, the genes regulating auxin synthesis had lower expression levels in flowering bulbs, but the genes involved in cytokinin, abscisic acid, and ethylene were commonly upregulated for flowering (Table 3, Figure 5).

In complex floral transition regulatory networks, the five regulatory pathways are not independent but often interact with each other to achieve their functions. Many key genes, transcription factors, and miRNAs have been reported to regulate flowering in different pathways, including integrin genes (*FT*, *SOC1*, *COL*, *LEAFY*, *AP1*, *AP2*), several transcription factor families, and miRNA156/172. For example, *COL* is regulated by many factors related to the meristem expression pattern, including temperature, light, transcription factors, and phytohormones (auxin, gibberellin, and abscisic acid) [41,50]. In the meristem of *L. chinensis* bulbs, 89 TFs families were identified, covering almost all families involved in plant growth and development. Most of the families were found to control the floral transition, including C2H2 [51], NAC [52], MYB [53], WRKY [54,55], AP2/ERF [56,57], NF-Y [58], and MADS-domain transcription factor [59]. Transcriptome and qRT-PCR analyses confirmed that different transcription factors have different expression patterns during bulb development and floral transition. For example, the MYB-related MYB1R1, NAC48, WRKY38, ERF12, and CRF1 have the highest expression levels in the four-year-old flowering bulbs, suggesting positive regulation of floral transition. In contrast, MYBAS2, NAC7, WRKY70, and ERF110 showed the lowest expression levels and may negatively regulate floral transition in *L. chinensis*. We also verified the expression of other core genes by qRT-PCR. *SPL14* and *SPL16* were shown to have the highest expression levels in the flowering bulbs. In the age pathway of flowering, SPL was shown to positively regulate the expression of miR172, and miR172 negatively regulates *AP2* (inhibitor of FT), finally leading to the regulation of plant flowering [4]. In this study, *AP2* was downregulated in the flowering bulbs, which is consistent with the mechanistic models of plant flowering by age pathway. Therefore, this study showed that the age is the main factor that regulates the flowering in *L. chinensis*, but the floral transition is a complex process involving physiological and biochemical changes, and it may be regulated by the sugar metabolism and hormone signal transduction pathways. Here, we provided abundant candidate differentially expressed TFs and genes associated with the bulb development and floral transition. Our data provide a genetic foundation for further research on the flowering regulatory mechanism of *Lycoris*.

## 5. Conclusions

In this study, we identified a large number of differentially expressed genes and transcription factors by RNA-Seq in *L. chinensis* bulbs of different ages. These DEGs were enriched in different GO and KEGG pathways in nonflowering and flowering bulbs, suggesting that they may regulate flowering by pathways including age, hormones, and vernalization in *Lycoris*. Starch and sucrose metabolism, anthocyanin biosynthesis, and catalytic activity are enhanced with increasing age and enlarged bulb size, and auxin is the main endogenous phytohormone involved in bulb growth and development. Once the bulb size is sufficient for flowering, the energy-metabolism-related genes maintain the high expression level, and the endogenous phytohormones (cytokinin, abscisic acid, and ethylene) also increase in flowering bulbs, causing some positive regulators (*SPL*, *COL,* and *AP1*) and early flowering genes in the *Lycoris* meristem to have high expression levels. These results suggest that sugar molecules may be the energy sources that regulate the floral signal transduction by connecting with phytohormone signaling in *Lycoris*. In these regulatory pathways, some key genes and TFs shown to contribute to plant flowering have been identified, but they have rarely been revealed in relation to *Lycoris* flowering regulation. Thus, our results provide an abundant genetic resource that can be used for further studies on flowering transition and time.

## Figures and Tables

**Table 1:** Summary of the RNA-Seq results in the bulb meristem of *L. chinensis*.

Sample	Raw Reads	Clean Reads	Mapped Reads (%)	Clean Bases (Gb)	Error (%)	Q30 (%)	GC Content (%)
P1_1	51364152	48050468	30055122 (62.55%)	7.21	0.02	91.77	46.39
P1_2	53461516	50651248	31333896 (61.86%)	7.6	0.02	91.73	45.94
P1_3	53097946	50340900	31405382 (62.39%)	7.55	0.02	91.69	45.97
P2_1	49727452	47050368	28607890 (60.80%)	7.06	0.02	91.74	45.79
P2_2	51056458	49258532	30434454 (61.79%)	7.39	0.02	91.59	46.37
P2_3	48469878	46229376	28821862 (62.35%)	6.93	0.02	92.08	45.88
P3_1	52193848	50317128	31317772 (62.24%)	7.55	0.02	92.03	45.85
P3_2	44948830	42822452	26548364 (62.00%)	6.42	0.02	91.88	46.02
P3_3	54775730	52580754	32810120 (62.40%)	7.89	0.02	91.66	46.34
P4_1	51261504	49312808	30595698 (62.04%)	7.4	0.02	92.90	45.65
P4_2	54918136	52885278	33199790 (62.78%)	7.93	0.02	92.64	45.96
P4_3	51105002	49211374	30602536 (62.19%)	7.38	0.02	91.76	46.05
P5_1	51373786	49198190	31129178 (63.27%)	7.38	0.02	92.86	46.17
P5_2	49618172	47438108	29441670 (62.06%)	7.12	0.02	93.01	46.04
P5_3	48961334	47145022	29901764 (63.43%)	7.07	0.02	92.75	47.05

P1–P3: One- to three-year-old bulbs that cannot flower, P4 and P5: nonflowering and flowering four-year-old bulbs. Three replications were conducted for each sample.

**Table 2:** The statistics of unigene annotation.

	Number of Unigenes	Percentage (%)
Annotated in NR	45,633	22.71
Annotated in NT	20,776	10.34
Annotated in KO	15,076	7.5
Annotated in Swiss-Prot	30,219	15.04
Annotated in PFAM	34,260	17.05
Annotated in GO	34,576	17.21
Annotated in KOG	8695	4.32
Annotated in all databases	4182	2.08
Annotated in at least one database	59,523	29.62
Total number of unigenes	200,902	100

**Table 3:** Key up- and downregulated genes involved in flowering in *Lycoris chinensis*.

Gene Description	Up or Down (P5 vs. P4)	Gene ID
squamosa promoter-binding-like protein 5/14/16	Up	c89274_g2, c94170_g2, c102363_g2
APETALA1-like MADS-box protein	Up	c97937_g1
protein early flowering 3-like	Up	c49522_g1
agamous-like MADS-box protein AGL14/AGL61	Up	c82036_g1, c52902_g1
zinc finger protein CONSTANS-LIKE 15-like (COL15)	Up	c106500_g4
zinc finger CCCH domain-containing protein 20-like/ 33-like/47-like/66-like	Up	c94525_g1, c106313_g3, c109144_g1, c109144_g2
AP2/ERF and B3 domain-containing transcription factor RAV1/RAV2-like	Up	c103183_g1, c93883_g1
ethylene-responsive transcription factor 2-like/4-like/ 12-like/1B-like/ ERF071-like/ERF096-like/ERF109-like	Up	c101524_g1, c108822_g1, c76676_g1, c96106_g1, c96726_g3, c104724_g1, c96726_g4
WRKY transcription factor 17/25/33/38/40/70	Up	c106688_g1, c106597_g3, c106597_g2, c92537_g1, c92627_g1, c110195_g2
transcription factor MYB4-like/MYB44-like/MYB108-like	Up	c106287_g1, c80135_g1, c102066_g2
transcription factor bHLH57	Up	c101985_g1
NAC domain-containing protein 8/45-like/48-like	Up	c95175_g1, c100874_g1, c97969_g1
cytokinin response regulator 1 protein	Up	c54926_g1
abscisic acid receptor PYL4-like	Up	c105195_g4
indole-3-acetic acid-amido synthetase GH3.1	Up	c93933_g1
gibberellin-regulated protein 6-like	Up	c62610_g1
energy-metabolism-related genes	Up	c110536_g3, c109813_g3, c91348_g1, c104769_g2, c71100_g1, c80903_g1, c94972_g2
U-box domain-containing protein 17-like/21-like/ 25-like/34-like	Up	c102443_g1, c100049_g1, c33997_g1, c103408_g1
F-box protein family-like protein	Up	c97221_g1
histone H2A/2B/H3/H4/4	Up	c102895_g2, c106587_g1, c107009_g1, c79513_g1, c110410_g1
terminal flower 1-like protein	Down	c76587_g1
protein UPSTREAM OF FLC-like	Down	c86645_g1
AP2-11	Down	c101346_g4
auxin-responsive protein IAA17-like	Down	c93453_g1
auxin-induced protein 22D-like	Down	c107268_g3
protein ethylene insensitive 3	Down	c109969_g1
transcription factor IBH1-like	Down	c93116_g2
NAC domain-containing protein 100-like	Down	c107517_g3
protein FAR1-RELATED SEQUENCE 5-like	Down	c82016_g1
E3 ubiquitin-protein ligase KEG	Down	c99481_g4
F-box protein At3g25750/At4g22060	Down	c72435_g1, c70935_g1

## Data Availability

The data were provided along with the manuscript as files in the Supplementary Materials. Raw reads were deposited in the NCBI database (https://www.ncbi.nlm.nih.gov/, accessed on 8 June 2022) under BioProject number PRJNA847051 (SRA accession: SRR19578267-SRR19578281).

## Supplementary Materials

The following supporting information can be downloaded at: https://www.mdpi.com/article/10.3390/biom12070899/s1, Figure S1: Principal component analysis of 469 DEGs in five samples of *Lycoris chinensis*. Each dot represents a sample, and colors code for stages P1–P5; Table S1: qRT-PCR premiers used in the study; Table S2: All unigenes identified in the study; Table S3: Differentially expressed genes in four comparison groups.
