# Supplementary material for: Transcriptome Analysis of Lycoris chinensis Bulbs Reveals Flowering in the Age-Mediated Pathway
Source: Biomolecules. 2022 Jun 27;12(7):899. doi: 10.3390/biom12070899 (PMC9312979; doi:10.3390/biom12070899)
Supplement: Supplementary file 1 [file biomolecules-12-00899-s001.zip › Figure S1.pdf]

Zhang et al., Transcriptome Analysis of *Lycoris chinensis* Bulbs Reveals Flowering in the Age-mediated Pathway

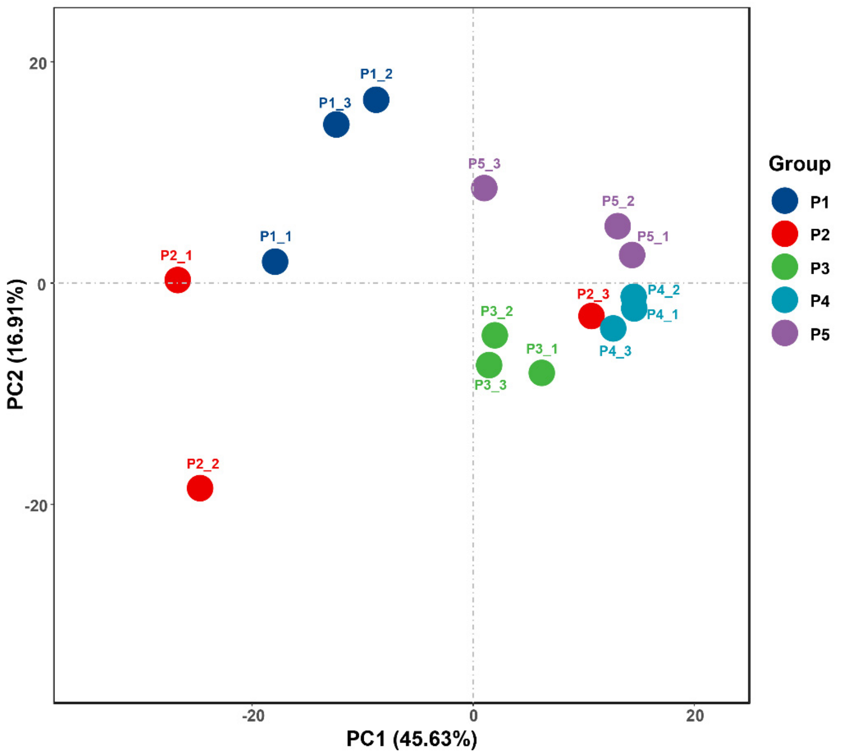

Figure S1: Principal component analysis of 469 DEGs in five samples of *Lycoris chinensis*. Each dot represents a sample, and colors code for stages P1-P5.
